# Alterations in the gut microbiota of AIDS patients with pneumocystis pneumonia and correlations with the lung microbiota

**DOI:** 10.3389/fcimb.2022.1033427

**Published:** 2022-10-21

**Authors:** Mingli Zhu, Sai Liu, Chenfei Zhao, Jinchuan Shi, Chaodan Li, Shisheng Ling, Jianghao Cheng, Wenkun Dong, Jiru Xu

**Affiliations:** ^1^ Department of Microbiology and Immunology, School of Basic Medical Sciences, Xi’an Jiaotong University, Xi’an, China; ^2^ Department of Microbiology, Hangzhou Xixi Hospital Affiliated to Zhejiang Chinese Medical University, Hangzhou, China; ^3^ Department of Clinical Laboratory, The First Hospital Affiliated to Zhejiang Chinese Medical University, Hangzhou, China; ^4^ Department of Infectious Diseases, Hangzhou Xixi Hospital Affiliated to Zhejiang Chinese Medical University, Hangzhou, China; ^5^ Research and Development Department, Assure Tech Institute of Medical Device, Hangzhou, China

**Keywords:** pneumocystis pneumonia, acquired immune deficiency syndrome, microbiome, gut-lung axis, CD4+ T cells

## Abstract

**Background:**

Due to the inability to be cultured *in vitro*, the biological characteristics and pathogenicity of *Pneumocystis jirovecii* remain unclear. Intestinal microflora disorder is related to the occurrence and development of various pulmonary diseases. This work explores the pathogenesis of pneumocystis pneumonia (PCP) in acquired immune deficiency syndrome (AIDS) patients from a microbiome perspective, to provide better strategies for the diagnosis, treatment, and prevention of PCP.

**Methods:**

Subjects were divided into three groups: human immunodeficiency virus (HIV)-infected patients combined with PCP, HIV-infected patients without PCP, and HIV-negative. Stool and bronchoalveolar lavage fluid (BALF) samples were collected, total DNA was extracted, and 16S rRNA high-throughput sequencing was performed using an Illumina MiSeq platform. PICRUSt and BugBase were used to predict microflora functions, and correlation analysis of intestinal and lung bacterial flora was conducted.

**Results:**

Compared with the HIV- group, prevotella and another 21 genera in the intestinal microbiome were statistically different in the HIV+ group; 25 genera including Escherichia-Shigella from HIV+PCP+ group were statistically different from HIV+PCP- group. The abundance of Genera such as Porphyromonas was positively or negatively correlated with CD16/CD56+ (μL). Compared with the HIV- group, identification efficiency based on area under the curve (AUC) >0.7 for the HIV+ group identified seven genera in the gut microbiota, including Enterococcus (total AUC = 0.9519). Compared with the HIV+PCP- group, there were no bacteria with AUC >0.7 in the lung or intestine of the HIV+PCP+ group. The number of shared bacteria between BALF and fecal samples was eight species in the HIV- group, 109 species in PCP- patients, and 228 species in PCP+ patients, according to Venn diagram analysis. Changes in various clinical indicators and blood parameters were also closely related to the increase or decrease in the abundance of intestinal and pulmonary bacteria, respectively.

**Conclusions:**

HIV infection and PCP significantly altered the species composition of lung and intestinal microbiomes, HIV infection also significantly affected intestinal microbiome gene functions, and PCP exacerbated the changes. The classification model can be used to distinguish HIV+ from HIV- patients, but the efficiency of bacterial classification was poor between PCP+ and PCP- groups. The microbiomes in the lung and gut were correlated to some extent, providing evidence for the existence of a lung-gut axis, revealing a potential therapeutic target in patients with HIV and PCP.

## Introduction

Acquired immune deficiency syndrome (AIDS) is a chronic infectious disease caused by human immunodeficiency virus (HIV) (Ghosn et al., 2018)) that destroys the function of the human immune defense system. Pneumocystis pneumonia (PCP) is a common opportunistic infection and ‘defining disease’ of AIDS (Atkinson et al., 2021). As a result of the widespread use of combined antiretroviral therapy (cART) and prophylactic treatment for PCP, the incidence of PCP in AIDS patients has declined substantially. It was once believed that PCP had become a rare disease in the post-AIDS period, but this was not the case. Recent studies have shown that PCP is still the main opportunistic infection among HIV-infected people in developed and developing countries, and a new problem among non-HIV immunodeficient patients (Esteves et al., 2014; Shibata and Kikuchi, 2019).

HIV infection may lead to dysregulation of the gut microbiota and immune activation. However, it is not known whether microbiota dysregulation is a cause or effect of immune alterations and disease progression, or whether it may modulate the risk of HIV infection (Lopera et al., 2021). The gastrointestinal mucosa is the core mucosal barrier and the largest source of CD4+ T cells in HIV patients. Although cART can effectively inhibit viral replication below the detection limit, mucosal immune dysfunction still occurs and cannot be fully recovered, resulting in an ectopic increase in intestinal microbes and their metabolites and systemic chronic inflammation, driving disease progression. The gut microbiota of HIV-infected individuals is typically characterized by an increase in Vibriosuccinis and Prevotella, and a decrease in Bacteroides (Lopera et al., 2021).

There is a close relationship between the intestine and the lung, including continuous cross-talk between the intestine and the lung mucosa through mesenteric and pulmonary lymph nodes. The gut regulates the immune responses of the lung through physical interaction, quorum sensing molecules, and synthesis of antimicrobial agents (Enaud et al., 2020). Metabolites of intestinal bacteria can reach other organs through the blood then regulate immune responses in distal mucosal sites such as lungs (Anand and Mande, 2018; Zhang et al., 2020). Changes in lung microbiota composition can affect the gut microbiota and *vice versa* (Dickson et al., 2016). Dysregulation of the intestinal flora leads to the occurrence of respiratory diseases (Fanos et al., 2020), and these changes can directly affect host defenses against acute respiratory pathogens, potentially risking death (Baradaran Ghavami et al., 2021).

Unlike many other fungi, *Pneumocystis jirovecii* (PJ) cannot grow sustainably in artificial media other than mammalian lungs, which has hampered studying the pathogenic mechanism of pneumocystis pneumonia. Previous studies have found that when HIV infection occurs, the respiratory flora changes (Bhadriraju et al., 2020). The intestinal microbiota can differ in AIDS-associated pneumonia patients, and these changes are significantly correlated with CD4 cell count, lung microbiota composition, and patient mortality (Shenoy et al., 2019). In mice, *P. murina* respiratory tract infection can significantly shift the diversity of the gut microbiota, modify the ability of the gut microbiota to metabolize carbohydrates, energy, and xenobiotics, and alter signal transduction pathways (Samuelson et al., 2016). However, changes occurring in the intestinal microbiota in PCP remain poorly understood. Therefore, based on the principle of the ‘gut-lung axis’, the present study focused on the relationship between the intestinal and lung microbiota in AIDS and PCP patients. We explored the mechanism of HIV and PCP from the perspective of microecology, with the intention of alleviating or preventing the occurrence of PCP by regulating the status of the microbiome.

## Materials and methods

### Participants

A total of 83 HIV-infected patients hospitalized in Hangzhou Xixi Hospital from 2017 to 2021 and eight healthy volunteers without HIV infection during the same period (controls; group C) were included in this study. HIV-infected patients were divided into two groups; 25 cases with PJ infection (group A) and 58 patients without PJ infection (group B). These patients were confirmed to be HIV-positive by chemiluminescence enzyme immunoassay (CLEIA) detection of anti-HIV-antibodies as well as P24 antigen using an HISCL HIV Ag+Ab Assay Kit (Sysmex Corporation, Kobe, Japan) and western blotting with an HIV Blot 2.2 MP Diagnostic Kit (MP Biomedicals Asia Pacific Pte., Ltd., Singapore). PCP was diagnosed by review of medical records, evaluation of radiological findings, and microbiological examination (positive for PJ by hexamine silver smear and/or real-time PCR). Only the first episode of PCP and one sample per patient were included in the analysis. Clinical data such as gender, age, diagnosis, and treatment, and laboratory data such as CD4+ T cell count, CD8+ T cell count, and CD4/CD8 ratio were collected. Patients with digestive disorders, gastrointestinal tract resection, significant dietary differences from other subjects, malignant tumors, serious diseases of the heart, brain, liver, and other important organs, and serious diseases of the blood and endocrine system were excluded.

### Sample collection

Fecal samples from HIV and non-HIV patients were collected at admission and processed in the laboratory within 4 h after collection. All fecal samples were dispensed in 2 ml Eppendorf tubes within 30 min, each tube containing 200 ± 20 mg, and immediately stored at -80°C until analysis. Through an electronic fiber bronchoscope, sterile normal saline was poured into the lungs and rinsed repeatedly, and 10−20 ml of bronchoalveolar lavage fluid (BALF) was collected, centrifuged at 3000 ×g for 10 min, and the sediment was immediately stored at -80°C.

### DNA extraction, PCR amplification, and DNA sequencing

Total fecal genome DNA samples were extracted from samples using a QIAamp DNA Stool Mini Kit (Qiagen, Valencia, CA, USA) according to the manufacturer’s instructions. DNA was extracted from BALF specimens using a TIANamp Micro DNA Kit (TIANGEN BIOTECH (BEIJING) CO., LTD, Beijing, China) according to the manufacturer’s instructions. After washing the spin column, 100 μl DNA was collected in a 1.5 ml tube by centrifugation. The DNA concentration in each sample was quantified using a Thermo NanoDrop 2000 spectrophotometer (Thermo Scientific, Wilmington, DE, USA). DNA integrity and size were monitored *via* 1% agarose gel electrophoresis. Extracted DNA was then directly used for PCR or stored at -20°C for later experiments.

Isolated bacterial genomic DNA was used as template for PCR amplification of the V3−V4 regions of the bacterial 16S rRNA gene. The 16S rRNA genes from the microbiota were amplified using bacterial primer set 341F (5’-CCTACGGGNGGCWGCAG-3’) and 805R (5’-GACTACHVGGGTATCTAATCC-3’). All PCR amplifications were carried out with 12.5 µl of Phusion Hot Start Flex 2X Master Mix (New England Biolabs, Ipswich, MA, USA), 0.2 µM of forward and reverse primers, and ~50 ng of template DNA. Thermal cycling involved an initial denaturation at 98°C for 30 s, followed by 35 cycles of denaturation at 98°C for 10 s, annealing at 54°C for 30 s, extension at 72°C for 45 s, and a final extension at 72°C for 10 min. PCR products were analyzed by 2% agarose gel electrophoresis, purified PCR products were quantified by a Qbit 2.0 Fluorimeter (Thermo Scientific, Waltham, MA, USA), and mixed in equal amounts. Sequencing libraries were generated using a TruSeq DNA PCR-Free Sample Preparation Kit (Illumina, San Diego, CA, USA) following the manufacturer’s recommendations, and index codes were added. Lastly, the library was sequenced on an Illumina MiSeq platform (Illumina) and 300 bp paired-end reads were generated.

### Microbiota analysis

According to the unique barcodes of samples, paired-end sequences were assigned to samples, barcode and primer sequences introduced in the library were removed, and matching ends were merged and read using FLASH software (http://www.cbcb.umd.edu/software/flash). According to Fqtrim (V0.94, https://github.com), raw read data were quality-filtered under specific filtering conditions to obtain high-quality clean labels. Chimeric sequences were filtered using Vsearch software (V2.3.4, https://github.com/torognes/vsearch). DADA2 (https://github.com/benjjneb/dada2) was used for demodulation to obtain the feature table and feature sequence. Then, according to SILVA (Release 132, https://www.arb-silva.de/) classifier, the relative abundance of each sample was used to normalize the feature abundance. Alpha-diversity and Beta-diversity were analyzed by QIIME2. The linear discriminant analysis (LDA) effect size (LEfSe) method was used to characterize taxa with statistical significance and biological relevance. Images were drawn by the R (V3.5.2) package. For species annotation, the feature classifier of QIIME2 was used for sequence alignment, with SILVA and NT-16S databases, but the SILVA database was mainly used for annotation. Based on Kyoto Encyclopedia of Genes and Genomes (KEGG) functional pathways, Phylogenetic Investigation of Communities by Reconstruction of Unobserved States (PICRUSt) was used to predict the functional composition of the intestinal and lung microbiome for each sample.

### Statistical analysis

Continuous variables are reported as the mean ± standard error of the mean (SEM), and statistical comparisons were made using one-way analysis of variance (ANOVA) followed by independent t-test. Non-normally distributed variables were expressed as interquartile ranges (IQRs), and comparisons were conducted using the Mann-Whitney U test, Kruskal-Wallis test, or the chi-square test. Receiver operating characteristic (ROC) analyses were performed to calculate the best cutoff points and area under the curve (AUC) for candidate biomarkers. For correlation analysis, Spearman’s rank test was performed. Statistical analysis was performed using SPSS version 22.0 (IBM Corporation, Armonk, NY, USA), and *p <0.05, **p <0.01, and ***p <0.001 were considered statistically significant.

## Results

### Study cohorts

There were 25 cases in the HIV+PCP+ group (group A), including 20 males and 5 females, aged 43.92 ± 13.40 years. There were 58 patients in the HIV+PCP- group (group B), including 47 males and 11 females, aged 45.90 ± 13.93 years. There were 8 cases in the HIV- (C) group, including 6 males and 2 females, aged 41.88 ± 17.66 years. All subjects were Han nationality, and there were no significant differences in clinical parameters between age and gender (*p >*0.05). All were born in Zhejiang, China, and had similar dietary structure.

### Richness, diversity, and composition of the gut microbiota

**Figure 1:**
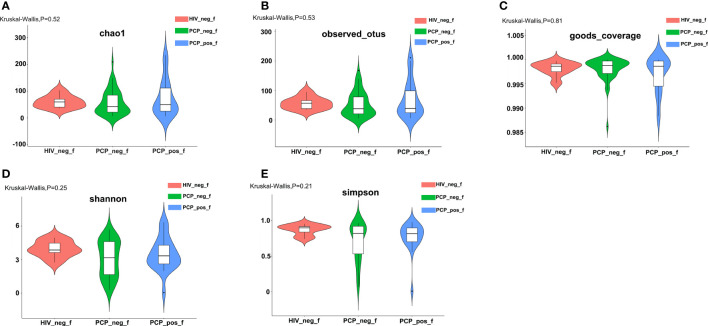
Comparison of α-diversity of the gut bacterial community among the three groups. **(A)** Comparison of the richness of the microbiota in the fecal samples among the three groups based on the Chao1 index (Kruskal-Wallis, p=0.52). **(B)** Comparison of the richness of the microbiota in the fecal samples among the three groups based on the Observed species index (Kruskal-Wallis, p=0.53). **(C)** Comparison of the OTU coverage of the microbiota in the fecal samples among the three groups based on the Goods_coverage index (Kruskal-Wallis, p=0.81). OTU: operational taxonomic unit. **(D)** Comparison of the fecal diversity of the microbiota among the three groups based on the Shannon index (Kruskal-Wallis, p=0.25). **(E)** Comparison of the fecal diversity of the microbiota among the three groups based on the Simpson index (Kruskal-Wallis, p=0.21). PCP_pos_f: human immunodeficiency virus (HIV)-infected patients combined with PCP (Pneumocystis pneumonia); PCP_neg_f: HIV-infected patients without PCP; HIV_neg_f: HIV-negative.

**Figure 2:**
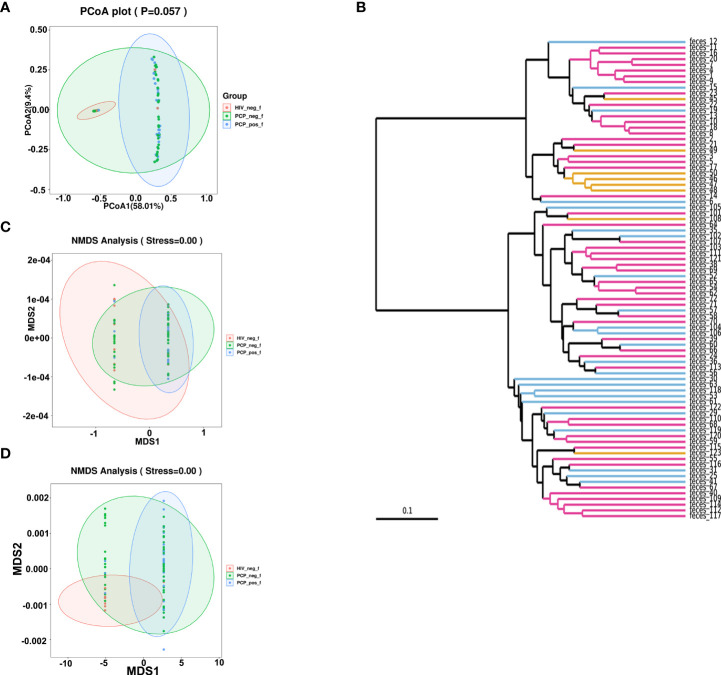
β-diversity analyses of fecal microbiomes of HIV+PCP+ group (PCP_pos_f), HIV+PCP- group (PCP_neg_f), and HIV- group (HIV_neg_f). The diversity of the samples was analyzed by **(A)** Principal coordinates analysis (PCoA) plot (unweighted_unifrac_pcoa). **(B)** Unweighted Pair Group Method with Arithmetic Mean (UPGMA) (unweighted_unifrac_feature). **(C)** Non-metric multidimensional scaling (NMDS) analysis (unweighted_unifrac_NMDS). **(D)** NMDS (weighted_unifrac_NMDS) analysis.

A total of 2,078,519 high-quality sequences were obtained from stool samples. The richness sparsity curve (Sobs index) was drawn to determine sequencing depth, and curves for each group were close to saturation, indicating that the sequencing depth was sufficient. There were no differences among the three groups (**Figures 1A–E**) in Chao1, Observed species, Goods_coverage, Shannon, Simpson, and other indices, which were used to reflect richness and uniformity. Beta (β)-diversity analysis showed that non-metric multidimensional scaling (NMDS; Weighted_UniFrac, Unweighted_UniFrac) results stress = 0.00 with a good representation. Unweighted pair group method with arithmetic mean (UPGMA) analysis showed that samples formed two large clusters, namely different intestinal types (**Figure 2**).

**Figure 3:**
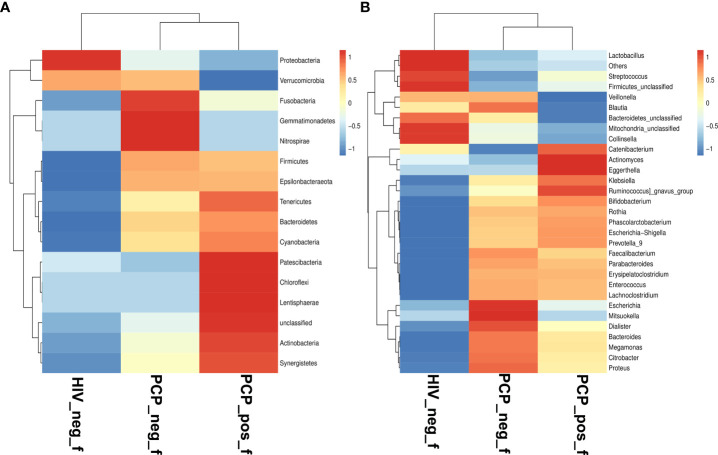
Comparison of gut microbiota at phylum and genus levels. **(A)** Relative abundance of the top 16 phyla in the fecal samples of the HIV+PCP+ (PCP_pos_f), HIV+PCP- (PCP_neg_f), and HIV- groups (HIV_neg_f,.Control). **(B)** Relative abundance of the top 31 genera in the fecal samples of the HIV+PCP+, HIV+PCP-, and HIV- groups. The gradient color from blue to red reflects the change in abundance from low to high.

According to the species abundance tables and annotation lists, the 30 most abundant species were selected to assess the relative abundance, and clustering of samples was analyzed according to distance (**Figure 3**).

### Gut microbiota differences between groups

**Figure 4:**
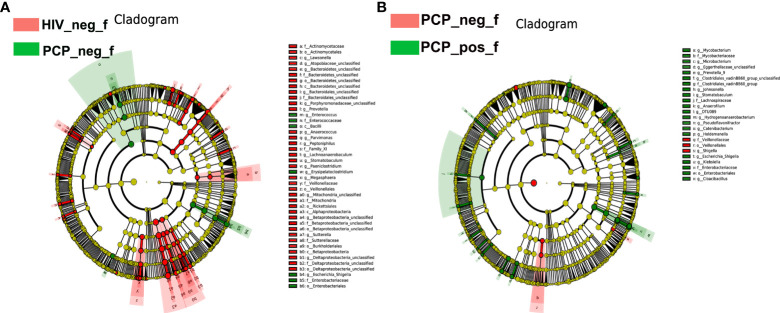
LEfSe diagram analysis of intestinal flora. Circles from the inside out indicate phylogenetic levels from phylum to genus. **(A)** LEfSe analysis of evolutionary clades of the gut microbiota in HIV+ and HIV- groups. **(B)** LEfSe analysis of evolutionary clades of gut microbiota in PCP+ and PCP- groups. HIV_neg_f: HIV- group; PCP_pos_f: HIV+PCP+ group; PCP_neg_f: HIV+PCP- group.

**Figure 5:**
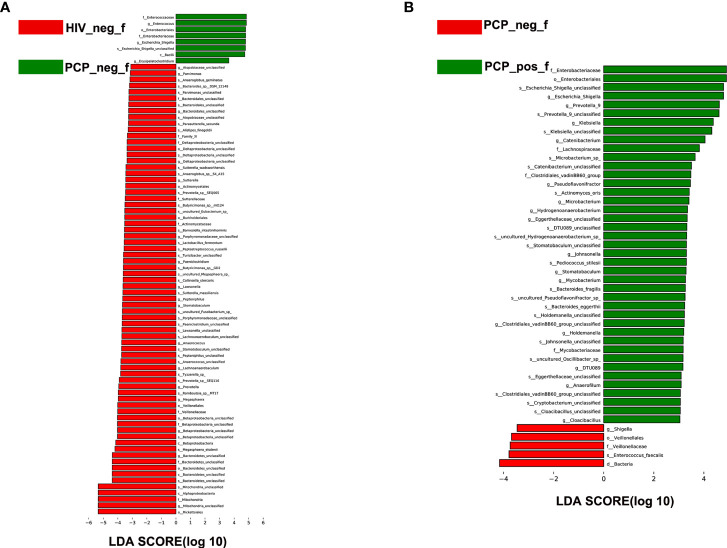
LEfSe analysis of intestinal flora. LEfSe identified the taxa with the greatest differences in abundance between groups. **(A)** LEfSe analysis of the intestinal microbiota in HIV+ and HIV- groups. **(B)** LEfSe analysis of the intestinal microbiota in PCP+ and PCP- groups. HIV_neg_f: HIV- group; PCP_pos_f: HIV+PCP+ group; PCP_neg_f: HIV+PCP- group.

To identify specific communities associated with HIV and PCP, we compared the gut microbiota composition between HIV+ and HIV-, and PCP+ and PCP- patients, using LEfSe analysis. For HIV+ and HIV-, LEfSe analysis revealed 31 discriminant features (LDA >3, *p <*0.05, **Figures 4A**, **5A**) at phylum (n = 0), family (n = 11), and genus (n = 20) levels. For HIV+PCP+ and HIV+PCP-, LEfSe analysis revealed 22 discriminant features (LDA >3, *p <*0.05, **Figures 4B**, **5B**) at phylum (n = 0), family (n = 5), and genus (n = 17) levels.

**Figure 6:**
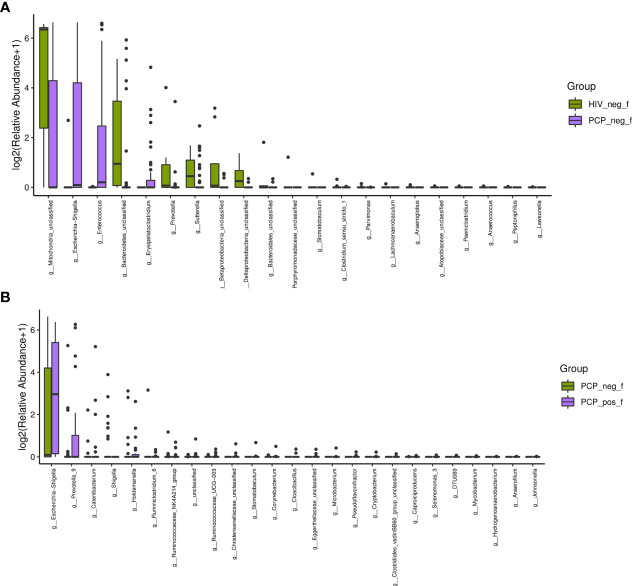
Characteristics of the gut microbiota composition among HIV+PCP+ group (PCP_pos_f), HIV+PCP- group (PCP_neg_f), and HIV- group (HIV_neg_f) at the genus level. **(A)** The differential genera are shown between HIV+PCP- and HIV- patients. **(B)** The differential genera are shown between HIV+PCP+ and HIV+PCP- patients.

There was no significant difference in the gut microbiota between B and C groups at the kingdom and phylum levels. At the genus level, 21 genera showed statistically significant differences, among which g_Betaproteobacteria_unclassified, g_Prevotella, g_Bacteroidetes_unclassified, g_Mitochondria_unclassified were increased (*p <*0.05); g_Enterococcus, g_Escherichia-Shigella, and g_Erysipelatoclostridium were decreased (*p <*0.05; **Figure 6A**). **Figure 6B** shows the difference between A and B groups. At the phylum level, P_unclassified was increased (mean 0.05% *vs*. 0.00%, *p* = 0.03). At the Genus level, 25 genera were statistically different, among which g_Prevotella_9, g_Holdemanella, g_Catenibacterium, and g_Escherichia-Shigella were increased (*p <*0.05).

### Relationship between intestinal microbial species and clinical indicators

**Figure 7:**
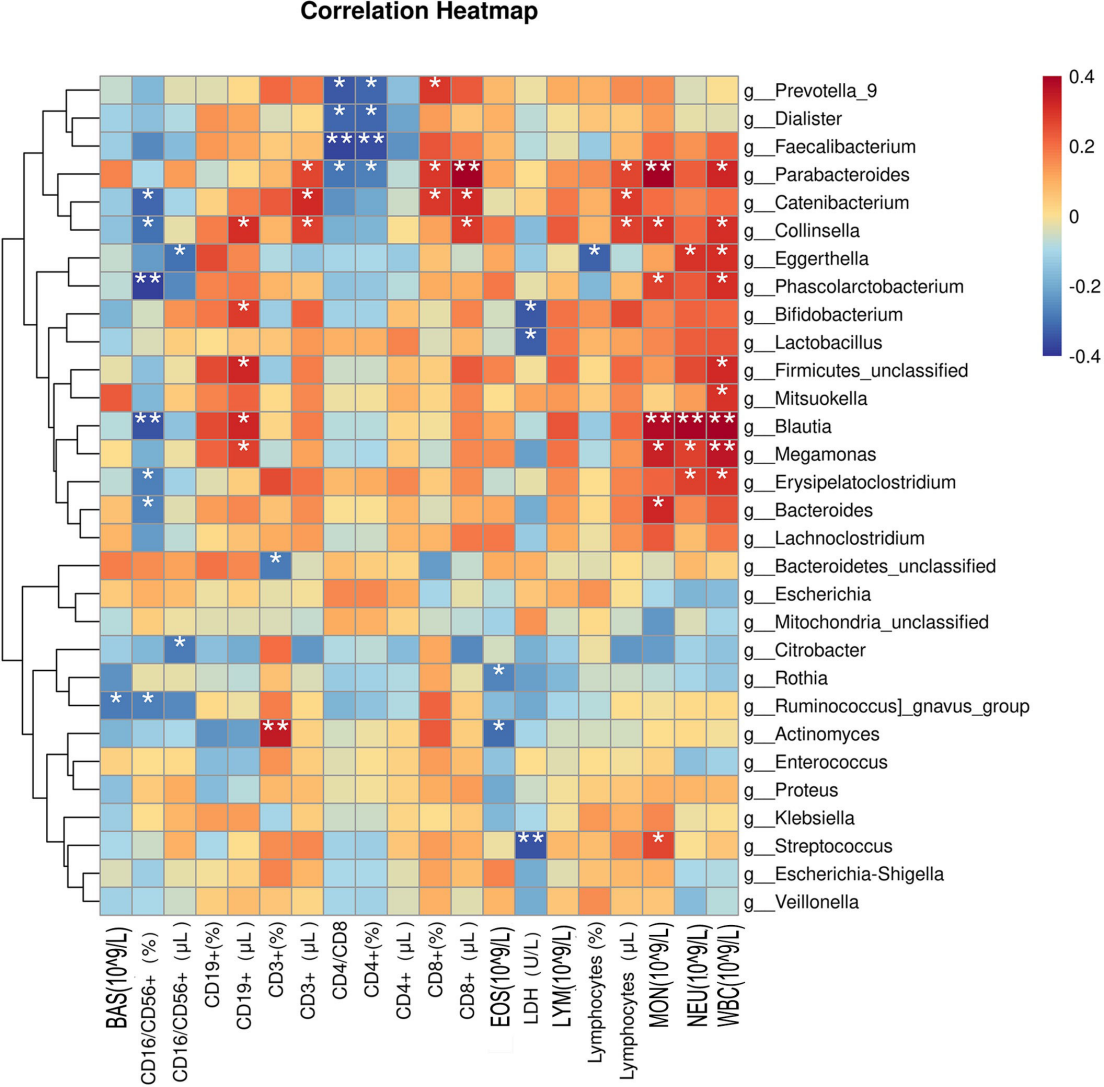
Correlations between clinical indicators and the top 30 microorganisms in the intestinal flora. BAS, basophil; CD, cluster of differentiation; EOS, eosinophil; LDH, lactic dehydrogenase; LYM, lymphocyte; MON, mononuclear cell; NEU, neutrophil; WBC, white blood cell. *p < 0.05 and ** p < 0.01 were considered statistically significant.

To investigate the classification ability of the gut microbiota of patients, correlation networks were generated using Spearman correlation analysis. Among the top 30 microbes, positive correlations were observed for g_Blautia, g_Megomonas, g_Parabacteroides, g_Firmiculta unclassified genera, g_Egerta, Koalbacter, g_Collinsella, g_Erysipelatoclostridium, and WBC (10^9^/L); g_Parabacteroides, g_Blautia, g_Megomonas, g_Bacteroides, g_Collinsella, g_Koala Bacillus, g_Streptococcus, and MON (10^9^/L); g_Parabacteroides, g_Catenibacterium, g_Collinsella, and CD8+ (μL); g_Blautia, g_Egteria, g_Megomonas, g_Erysipelatoclostridium, and NEU (10^9^/L); g_Actinomycetes and CD3+(%); g_Firmicutes unclassified genera, g_Blautia, g_Collinsella, g_Bifidobacterium, g_Megomonas, and CD19+ (μL), g_Catenibacterium g_Collinsella, g_Parabacteroides, and CD3+ (μL); g_Catenibacterium, g_Parabacteroides, g_Prevotella 9, and CD8+(%); g_Catenibacterium, g_Collinsella, g_Parabacteroides, and Lymphocytes (μL). Meanwhile, negative correlations were observed for g_Colobacter, g_Blautia, g_Catenibacterium, g_Collinsella, g_Ruminococcus active group, g_Erysipelatoclostridium, g_Bacteroides, and CD16/CD56+ (%); g_Faecalis, g_Prevotella 9, g_Dialister, g_Parabacteroides, and CD4/CD8; g_Faecalis, g_Dialister, g_Prevotella 9, g_Parabacteroides, and CD4+(%); g_Streptococcus, g_Bifidobacterium, g_Lactobacillus, and LDH (U/L); g_Egteria and Lymphocytes (%); g_Actinomycetes, g_Roche, and EOS (10^9^/L); g_Egteria, g_Citrobacter, and CD16/CD56+ (μL); g_Ruminococcus active group and BAS (10^9^/L); and g_Bacteroidetes unclassified genera and CD3+(%) (**Figure 7**).

### Identification of HIV and PCP based on intestinal flora

**Figure 8:**
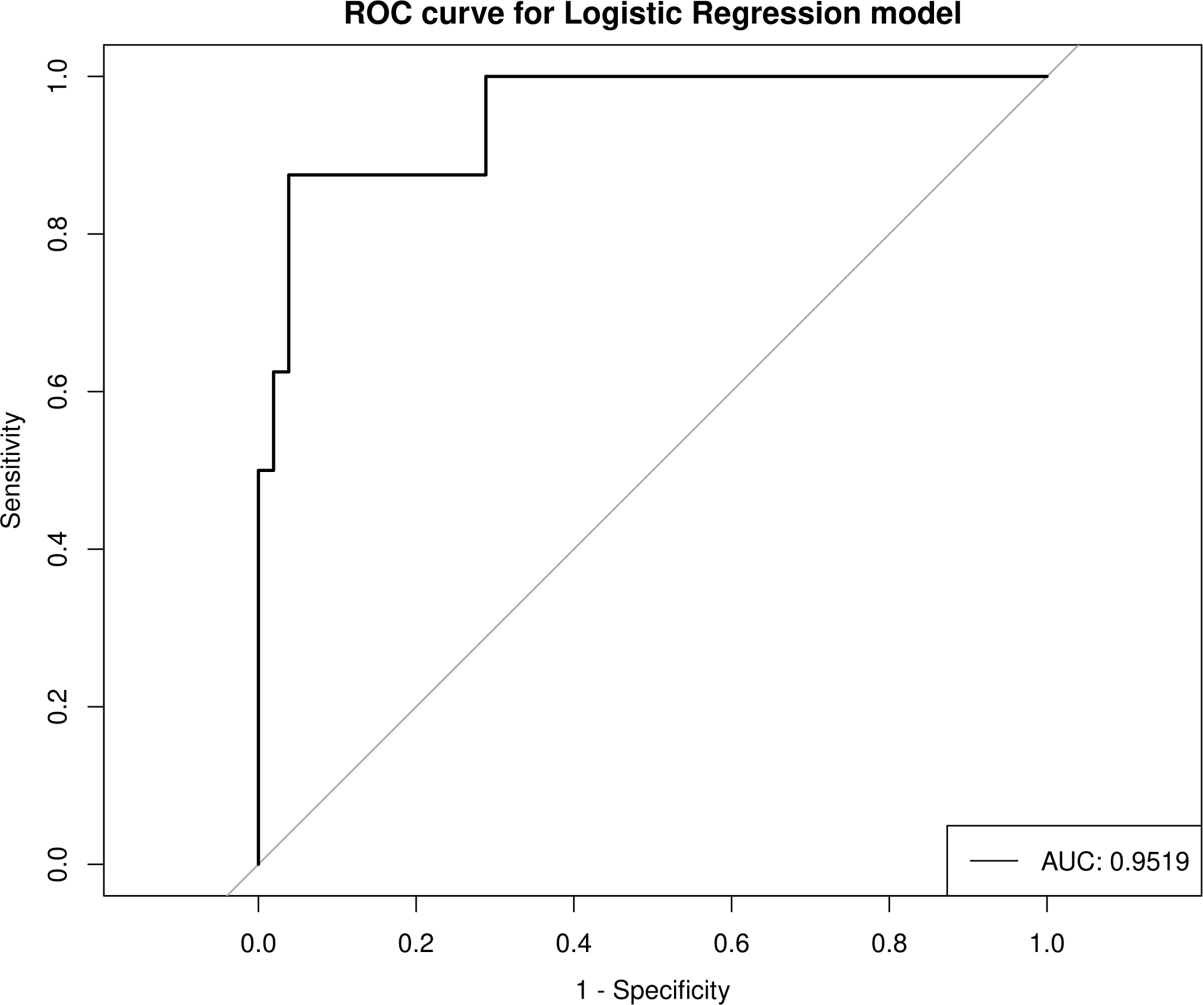
Prediction model for HIV+ and HIV- discrimination based on seven combined genera of gut microbiota. AUC, area under the curve; ROC, receiver operator characteristic curve.

In order to explore the discrimination ability of the intestinal microbiota for HIV and PCP, candidate bacteria were selected according to LDA value, and based on the AUC value. Finally, the model with the best performance was selected as the final model. When differentiating between HIV+ and HIV- patients, those with AUC >0.7 were g_Mitochondria_unclassified (0.7596), g_Escherichia-Shigella (0.7284), g_Enterococcus (0.7788), g_Bacteroidales_unclassified (0.7644), g_Prevotella (0.7115), g_Betaproteobacteria_unclassified(0.7212), and g_Deltaproteobacteria_unclassified (0.7404), and the total AUC reached 0.9519 (**Figure 8**), indicating a significant difference between HIV+ and HIV- control groups. These results confirmed that a classification model based on the gut microbiota could distinguish HIV+ patients from HIV-patients. For the differential intestinal flora of PCP+ and PCP- patients, no bacterial genera with AUC >0.7 were identified.

### Function prediction for gut microbiota in HIV and PCP patients

**Figure 9:**
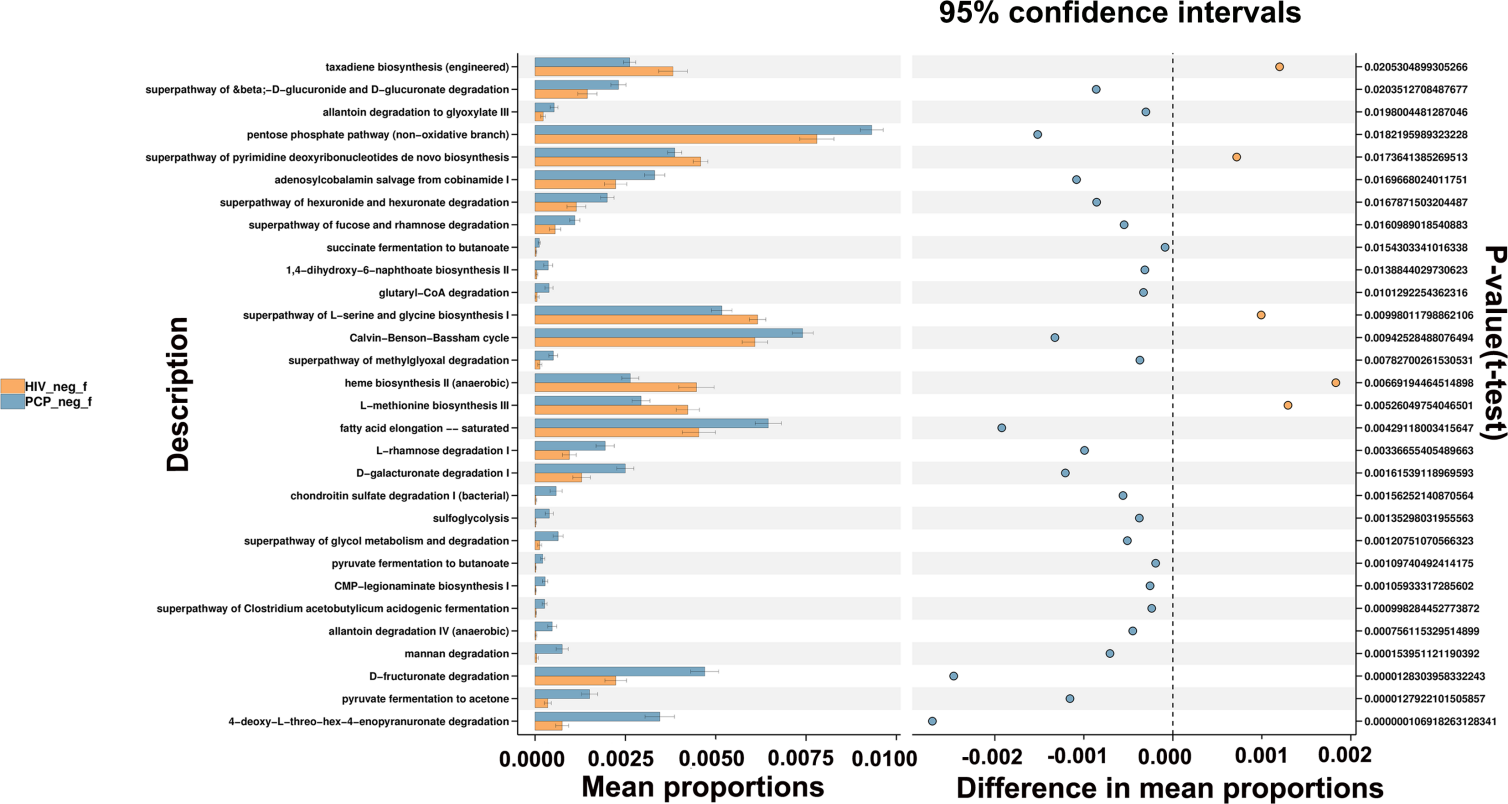
The difference in the KEGG pathways of gut microbiota between HIV+PCP- and HIV- patients. HIV_neg_f: HIV- group; PCP_neg_f: HIV+PCP- group.

**Figure 10:**
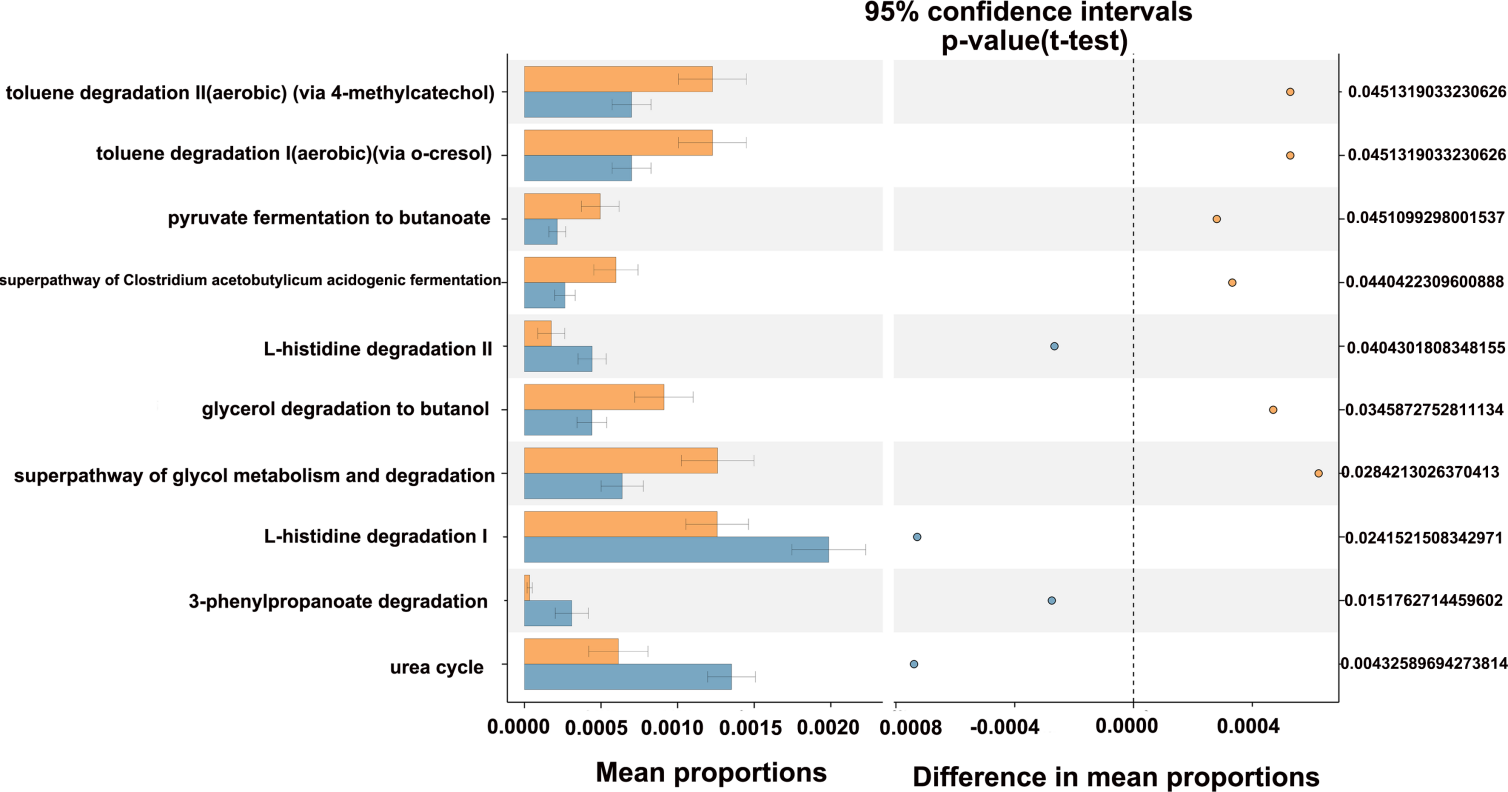
The difference in the KEGG pathways of gut microbiota between HIV+PCP+ and HIV+PCP- patients. PCP_neg_f: HIV+PCP- group; PCP_pos_f: HIV+PCP+ group.

The potential metabolic function of gut microbiota influenced by HIV infection and PCP was further predicted using the Phylogenetic Investigation of Communities by Reconstruction of Unobserved States (PICRUSt2) analysis. According to the functional prediction map of the metabolic pathway, it was found that KEGG metabolic pathways involved pentose phosphate pathway, Calvin-Benson-Bassham cycle, fatty acid elongation saturated, D-fructuronate degradation, etc. between HIV+PCP- and HIV- patients (**Figure 9**), and L-histidine degradation, urea cycle, toluene degradation, etc. between HIV+PCP+ and HIV+PCP- patients (**Figure 10**). Based the paired Mann-Whitney-Wilcoxon test, compared with HIV-, anaerobic relative abundance in HIV+PCP- decreased, while the gene function of the contains_mobile_elements increased.

### Comparison of lung and intestinal microbiome composition

**Figure 11:**
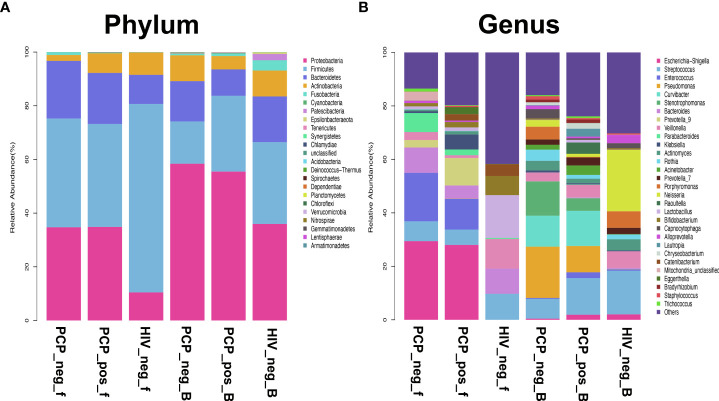
Comparison of phylum and genus level microbiota in the gut and lung. HIV_neg_f: gut microbiomes of HIV- group; PCP_pos_f: gut microbiomes of HIV+PCP+ group; PCP_neg_f: gut microbiomes of HIV+PCP- group; HIV_neg_B: lung microbiomes of HIV- group; PCP_pos_B: lung microbiomes of HIV+PCP+ group; PCP_neg_B: lung microbiomes of HIV+PCP- group. **(A)** Comparison of phylum level microbiota in the gut and lung. **(B)** Comparison of genus level microbiota in the gut and lung.

Sequencing analysis at the Phylum level showed that Firmicutes were the most abundant component in the intestinal tract of HIV-infected patients (70.19%), and the relative abundance of Firmicutes in the lung was also high (30.48%; the most abundant Proteobacteria was 35.97%). Firmicutes was the most abundant component in the intestinal tract (40.54%) in HIV+ (PCP-) patients, and the relative abundance of Firmicutes in the lung was also high (15.75%; the most abundant Proteobacteria was 58.42%). Firmicutes was the most abundant component in the intestine of PCP+ patients (38.33%), and the relative abundance of Firmicutes in lungs was also high (28.24%; the abundance of Proteobacteria was 55.48%). Genus-level sequencing analysis showed that Streptococcus, Enterococcus, and Veillonella were the most abundant genera in the gut and lung (**Figure 11**).

### Common species in lung and gut microbiomes

**Figure 12:**
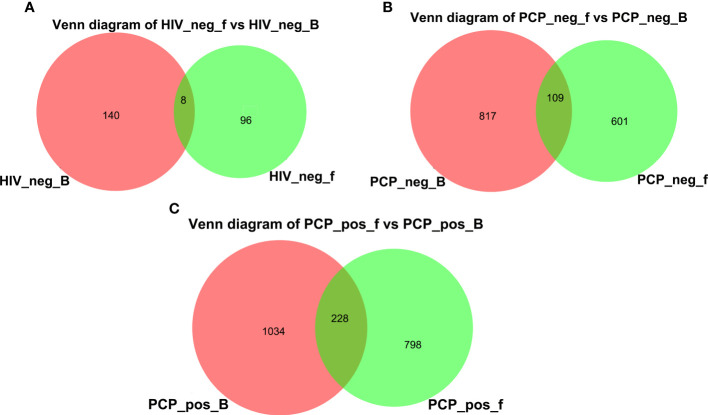
Venn diagram of lung and gut microbiomes. **(A)** Venn diagram of lung and gut microbiomes in HIV- patients. **(B)** Venn diagram of lung and gut microbiomes in HIV+PCP- patients. **(C)** Venn diagram of lung and gut microbiomes in HIV+PCP+ patients. HIV_neg_B: lung microbiomes of HIV- group; HIV_neg_f: gut microbiomes of HIV- group; PCP_neg_B: lung microbiomes of HIV+PCP- group; PCP_neg_f: gut microbiomes of HIV+PCP- group; PCP_pos_B: lung microbiomes of HIV+PCP+ group; PCP_pos_f: gut microbiomes of HIV+PCP+ group.

A Venn diagram was drawn to show bacteria shared with or unique to BALF and feces samples (**Figure 12**). The diagram shows revealed eight species in HIV- BALF and stool samples, 109 species in HIV+PCP- patients, and 228 species in HIV+PCP+ patients. Therefore, HIV and PCP infection could lead to an increase in co-bacterial genera in the lung and intestinal flora of patients.

### Correlations between clinical indicators and intestinal or pulmonary microbiota

As can be seen from **Table 1**, alterations in various clinical indicators are closely related to an increase or decrease in the abundance of various bacterial genera in the intestinal and/or respiratory microbiota. Therefore, in addition to previously reported metabolites such as fatty acids, the intestinal and lung microbiota can also be related to other factors such as CD4+ T cells, CD4/CD8 ratio, and WBC.

**Table 1:** Correlations between clinical indicators and intestinal or pulmonary microbiota.

	BALF		Feces	
	Positive correlation	Negative correlation	Positive correlation	Negative correlation
CD16/CD56+ (μL)	g_Porphyromonas, g_Neisseria			g_Eggerthella, g_Citrobacter
Lymphocytes (μL)	g_Neisseria, g_Porphyromonas		g_Catenibacterium, g_Collinsella, g_Parabacteroides	
Lymphocytes (%)	g_Neisseria	g_Klebsiella		g_Eggerthella
CD3+ (μL)	g_Neisseria, g_Porphyromonas		g_Catenibacterium, g_Collinsella, g_Parabacteroides	
BAS (10˄9/L)	g_Sphingomonas, g_Bradyrhizobium, g_Curvibacter, g_Ralstonia	g_Mitochondria_unclassified		g_Ruminococcus_gnavus_group
CD19+ (μL)	g_Haemophilus, g_Neisseria		g_Firmicutes_unclassified, g_Blautia, g_Collinsella, g_Bifidobacterium, g_Megamonas	
NEU (10˄9/L)		g_Pelomonas, g_Curvibacter, g_Methylobacterium, g_Sphingomonas, g_Bradyrhizobium, g_Sediminibacterium, g_Acinetobacter	g_Blautia, g_Eggerthella, g_Megamonas, g_Erysipelatoclostridium	
WBC (10˄9/L)		g_Curvibacter, g_Pelomonas, g_Methylobacterium	g_Blautia, g_Megamonas, g_Parabacteroides, g_Firmicutes_unclassified, g_Eggerthella, g_Phascolarctobacterium, g_Collinsella, g_Mitsuokella	g_Erysipelatoclostridium, g_Sutterella
MON (10˄9/L)			g_Parabacteroides, g_Blautia, g_Megamonas, g_Bacteroides, g_Collinsella, g_Phascolarctobacterium, g_Streptococcus	
CD8+ (μL)			g_Parabacteroides, g_Catenibacterium, g_Collinsella	
CD3+ (%)			g_Actinomyces	g_Bacteroidetes_unclassified
CD8+ (%)			g_Catenibacterium, g_Parabacteroides, g_Prevotella_9	
CD16/CD56+ (%)				g_Phascolarctobacterium, g_Blautia, g_Catenibacterium, g_Collinsella, g_Ruminococcus_gnavus_group, g_Erysipelatoclostridium, g_Bacteroides
CD4/CD8				g_Faecalibacterium, g_Prevotella_9, g_Dialister, g_Parabacteroides
CD4+ (%)				g_Faecalibacterium, g_Dialister, g_Prevotella_9, g_Parabacteroides
LDH (U/L)				g_Streptococcus, g_Bifidobacterium, g_Lactobacillus
EOS (10˄9/L)				g_Actinomyces, g_Rothia

g_: Genus:; BALF, bronchoalveolar lavage fluid; CD, cluster of differentiation; BAS, basophil; NEU, neutrophil; WBC, white blood cell; MON, mononuclear cell; LDH, lactic dehydrogenase; EOS, eosinophil.

## Discussion

Accumulating evidence shows that the gut microbiota plays a crucial role in human health. Changes in the composition and function of the microbial flora are associated with body growth, development, infection, autoimmune processes, metabolic diseases, and neoplastic status of colon, rectal, breast, liver, and other tissues. The gastrointestinal tract is the reservoir of CD4+ T cells, and one of the main immune centers. The gut microbiota has an important impact on immune response pathways and cell networks (Li et al., 2019). The human microbiome is thought to be the main regulator of the immune system in healthy and diseased states. A previous study found that the gut microbiota may affect lungs through the gut-lung axis, with implications for immune responses and homeostasis in the respiratory tract. In the present study, we found that the species composition and function of the intestinal microbiota in AIDS and PCP patients can change regularly, this is related to the lung microbiota through a variety of factor, and it plays an important role in the occurrence and development of AIDS and PCP.

AIDS is caused by HIV infection, and is related to gastrointestinal diseases, systemic immune activation, and alterations in the intestinal microbiota (Zilberman-Schapira et al., 2016). The gastrointestinal tract is the main target of HIV attack. HIV infection is also characterized by changes in the composition and function of the gut microbiota (Zilberman-Schapira et al., 2016). Most studies have found that HIV infection is associated with intestinal dysregulation and changes in microbiota diversity, manifested by increased numbers of Enterobacteriaceae and Enterococciaceae pathogens, as well as enrichment of Prevotella (Yu et al., 2014; Mutlu et al., 2014; Dinh et al., 2015; Li et al., 2019; Schluter et al., 2020; Do Nascimento et al., 2021). One study enrolled 33 HIV patients and 35 healthy controls (HCS) in a southern Chinese population and showed that compared with healthy individuals, HIV infection-related disorders were mainly manifested as a decrease in α-diversity and the level of Bacteroidetes, and an increase in Proteobacteria (Zhou et al., 2018). After treatment, HIV-infected fecal microbiota imbalance was partially restored. In our preliminary study, we found no significant difference in α-diversity among groups. Untreated and healthy control groups showed no obvious differences in β-diversity. Compared with the control group, the untreated HIV-infected group had reduced species diversity and significantly reduced beneficial colonies. Compared with the untreated HIV-infected group, the intestinal microbiota of the HIV-infected group receiving cART treatment but without negative viral load was further disordered, and the species diversity was further decreased. After cART, the species diversity of HIV-infected patients with negative viral load was slightly increased, but an improvement in the intestinal microbiota was not obvious. Some other studies have drawn similar conclusions, finding that cART cannot continuously restore the gut microbiota (Li et al., 2016).

HIV infection leads to changes in the intestinal microbiota, which is related to immune activation and chronic inflammation. The loss of T helper cells (Th) induced by HIV infection can lead to damage of the intestinal barrier, resulting in intestinal immune abnormalities and dysregulation (Ashuro et al., 2020). Some metabolic changes of the microbiota are related to oxidative stress (Vázquez-Castellanos et al., 2015). Decreased interleukin 10 (IL-10) and IL-1R levels are in turn associated with increased activation of CD8+ T cells (Dillon et al., 2014; Rinaldi et al., 2021), which may promote infection in HIV-exposed individuals. The presence and abundance of beneficial bacteria in the gut microbiota can regulate the development and response of the immune system, reduce the activation level, promote the integrity and function of the intestinal mucosa, and thus prevent infection by intestinal pathogens and sexually transmitted viruses such as HIV-1 (Kuebler et al., 2016). Some researchers used flow cytometry to detect the activation of CD4 and CD8 T cells and the Th17 frequency of lamina propria lymphocytes in five different sites of the intestine, and real-time fluorescence quantitative PCR was used to measure the expression levels of IFNβ, IFNAR1, and MxA genes in lamina propria lymphocytes. The results showed that the fecal microbial flora of HIV-1-infected men was significantly unbalanced, especially Faecalibacterium, Prevotella, Alistipes, and Bacteroides (Pinacchio et al., 2020).

In the present study, there were no statistically significant differences at Kingdom and Phylum levels between HIV+ and HIV- groups. At the Genus level, 21 genera were statistically different, including increases in g_Betaproteobacteria_unclassified, g_Prevotella, g_Bacteroidetes_unclassified, g_Mitochondria_unclassified (*p <*0.05), and decreases in g_Enterococcus, g_Escherichia-Shigella, g_Erysipelatoclostridium (*p <*0.05). A total of 25 genera were statistically different between HIV+PCP+ and HIV+PCP- groups, including increases in g_Prevotella_9, g_Holdemanella, g_Catenibacterium, and g_Escherichia-Shigella (*p <*0.05). An increase in Prevotella in HIV infection has been reported many times, and further demonstrated in this work. We also found an increase of Prevotella in PCP. Previous studies found that the abundance of Prevotella is positively correlated with the frequency of CD38 or HLA-DR expression, and both CD38 and HLA-DR expression in CD4 T cells (*p <*0.05), and activated CD8 T cells displayed the same trend. Prevotella levels and IFN-I gene (*p <*0.05) and Th17 cell (*p <*0.05) frequency were negatively correlated. These results suggest that Prevotella enrichment may affect the IFN-I pathway and T cell responses in the intestinal mucosa of HIV-1-infected patients, thereby leading to immune dysfunction (Pinacchio et al., 2020). AIDS may lead to changes in the intestinal microbiota, PCP can lead to further changes, and enrichment of Prevotella may be related to the pathogenesis of PCP.

PCP is a common opportunistic fungal infection in AIDS patients that can cause poor prognosis and even death. There is increasing evidence that the gut microbiota is essential for host defenses against fungal pathogens (Samuelson et al., 2016). However, few studies have explored the gut microbiota of PCP patients. In one study, C57BL/6 mice were endotracheally inoculated with viable *P. murina* and sacrificed after 7 and 14 days for microbiota analysis. The results showed that the diversity of the intestinal microbial population changed significantly after respiratory infection with pneumocystis. In contrast to immunocompetent mice infected with *P. murina*, the intestinal microbiota of CD4+ T cell null mice was significantly altered, suggesting that the loss of CD4+ T cells may also affect the intestinal microbiota of PCP. In functional prediction, it was found that PCP significantly altered the ability of intestinal microorganisms to respond to carbohydrate, energy, and heterogenic metabolism, and signal transduction pathways (Samuelson et al., 2016). In the respiratory tract, Pneumocystis can coexist with other pathogenic microorganisms, and infection can change the local and systemic environment, which may affect the host microbiota locally and at a distance. In fact, in the mouse model described above, the gut microbiota can be altered by the presence of Pneumocystis, supporting the objective existence of the gut-lung axis.

The gut-lung axis refers to the physiological and pathological interaction between lung and intestine in the process of disease occurrence. Changes in the microbial composition of the lungs can affect the gut microbiome and *vice versa*. The lung-gut axis is potentially involved in the pathogenesis of many diseases (Cillóniz et al., 2019; Ashuro et al., 2020). There are many related mechanisms between lung and intestine, including microbiome translocation. The lower digestive tract may be a possible source of bacterial flora in the lungs of critically ill patients (Liu et al., 2022). Bacterial transfer from gut to lung due to possible barrier dysfunction has been demonstrated in sepsis and acute respiratory distress syndrome (Depner et al., 2020; Saint-Criq et al., 2021). Fungi are also an important part of the gut microbiota, and parenteral function is the most prominent feature of intestinal fungi. Intestinal fungi are very important in the regulation of intestinal, lung, liver, kidney, pancreas and brain functions, and have good application prospects in the mitigation/treatment of human diseases (Wu et al., 2021).

In the present study, Firmicutes were found to be the most abundant component in the intestines of HIV+ and PCP+ groups, Firmicutes and Proteobacteria were the two most abundant phyla in lungs, and the abundance of Firmicutes gradually decreased. Sequencing analysis at the genus level showed that Streptococcus, Enterococcus, and Veillonella were the most common genera in the intestine and lung. Venn diagram analysis identified eight species in non-HIV BALF and stool samples, 109 species in HIV+PCP- patients, and 228 species in the HIV+PCP+ patients, indicating that HIV and PCP infection may increase the abundance of co-bacterial genera in patient lungs and the intestinal microbiota through the connection between lung and intestine.

The gut is essential for immune responses and homeostasis in the respiratory tract (Dumas et al., 2018). The microbiota in the lung and intestine can be associated with immunity through microbial-related molecules and metabolites (Dickson et al., 2016). The gut microbiota and its associated molecules and metabolites are transferred through the circulatory system from the intestinal lumen to various organs (liver, brain, lung), and subsequently induce tissue-specific local immune responses. In the lung, short-chain fatty acid (SCFA)-induced bone marrow cells migrate to the lung, resulting in pulmonary immune changes. A Clostridium catechol-derived product, deaminotyrosine, regulates type I IFN signaling, and exposure to different pulmonary microorganisms (e.g., Pseudomonas and Lactobacillus) is associated with the enhanced Th17 responses (Wu et al., 2021).

The present study found that changes in various clinical indicators of HIV and PCP were closely related to an increase and/or decrease in the abundance of various bacterial genera in the intestinal microbiota and respiratory microbiota. For example, compared with non-HIV controls, for HIV patients, CD4+(%) was positively correlated with Lactobacillus, Staphylococcus, Androgectomyces, Klebsiella, Actinobacter, Streptococcus, Alloprevotella, Haemophilus, Akkermansia, and Enterococcus, but negatively correlated with Reyranella, Mycobacterium, Oligotrophomonas, and Methylbacillus in lung, and positively correlated with intestinal β-proteobacteria unclassified. Compared with PCP-, CD4+(%) was positively correlated with pulmonary Roving cocci, negatively correlated with intestinal Ruminococcaceae_UCG-003, Prevoella 9, Christensenellaceae_unclassified, Eggerthellaceae_unclassified, and g_Caproiciproducen in PCP. Previous research established that the microbiome generally affects the body’s immune system in two ways; by producing microbe-related molecules, or *via* metabolites produced by the microbiome (Dickson et al., 2016). In addition, metabolites of the gut microbiota are associated with immune-mediated pathways that regulate intestinal Niemann-pick C1-like 1 expression (Enaud et al., 2020). The abundance of PJ is positively correlated with a decrease in total leukocyte count and immune deficiency (Yan et al., 2021). Interestingly, in the present study, a variety of bacteria showed similar associations with different immune cells in the lung and gut. These results indicate that in addition to reported factors such as microbial translocation and fatty acids, the intestinal and pulmonary microbiota can also be linked by many influencing factors such as CD4+ T cells.

Respiratory diseases involve the coordinated development of microbes in the gut and lung, related exogenous and endogenous factors, the gut-lung axis, and its dysregulated mechanism of action. Although our current understanding of the gut-lung axis is in its infancy, several strategies related to the gut microbiota have been employed to treat and prevent lung diseases (Budden et al., 2017; Tan et al., 2020). Exploring the microbiota characteristics of diseases related to the lung-intestinal axis is helpful for diagnosis and prognosis. In addition, gut specimens are more readily available than lung specimens in most cases, and if we can understand the correlation between the lung and gut microbiomes, it may be possible to infer the composition of the lung microbiome from the gut to optimize the clinical diagnostic process. In future work, the sample size should be expanded to further verify the correlations between certain pathogens and immunity. Using this approach, the presence of certain pathogens could be determined from changes in the number of immune cells, thereby improving diagnostic accuracy for certain pathogens.

## Conclusion

In summary, 16S rRNA high-throughput sequencing of different gut and lung samples showed that HIV infection and PCP significantly altered the species composition of lung and intestinal microbiomes, and the bacterial community disorder for HIV infection was greater than for PCP. HIV infection also significantly affected the gene functions of the intestinal microbiomes, and PCP further promoted these changes. We found close correlations between different microorganisms and clinical indicators. The classification model can be used to distinguish HIV from HIV- patients, but the efficiency of bacterial classification was poor between PCP+ and PCP- groups. The microbiomes in the lung and gut were correlated to some extent, providing evidence for the existence of the lung-gut axis, which might play an important role in patients. In addition, the microbiome was correlated with immunity, indicating a potential therapeutic target in patients with HIV and PCP.

## Data availability statement

The data presented in the study are deposited in https://www.ncbi.nlm.nih.gov/, accession number PRJNA882622.

## Ethics statement

The studies involving human participants were reviewed and approved by Hangzhou Xixi Hospital, Affiliated to Zhejiang Chinese Medical University. The patients/participants provided their written informed consent to participate in this study.

## Author contributions

MZ contributed to investigation, methodology, data curation, writing the original draft, and resources. SL, CZ, JS, CL, SSL, and JC contributed to data curation. WD and JX contributed to supervision, resources, and reviewing the manuscript. All authors contributed to the article and approved the submitted version.

## Funding

This work was supported by the Hangzhou Biological Medicine and Health Industry Development Support Science and Technology Project (2021WJCY363) and the Science and Technology Project from Health Commission of Hangzhou (2017Z09).

## Conflict of interest

The authors declare that the research was conducted in the absence of any commercial or financial relationships that could be construed as a potential conflict of interest.

## Publisher’s note

All claims expressed in this article are solely those of the authors and do not necessarily represent those of their affiliated organizations, or those of the publisher, the editors and the reviewers. Any product that may be evaluated in this article, or claim that may be made by its manufacturer, is not guaranteed or endorsed by the publisher.
